# *Lysinibacilli*: A Biological Factories Intended for Bio-Insecticidal, Bio-Control, and Bioremediation Activities

**DOI:** 10.3390/jof8121288

**Published:** 2022-12-08

**Authors:** Qazi Mohammad Sajid Jamal, Varish Ahmad

**Affiliations:** 1 Department of Health Informatics, College of Public Health and Health Informatics, Qassim University, Al Bukayriyah 52741, Saudi Arabia; 2 Health Information Technology Department, The Applied College, King Abdulaziz University, Jeddah 21589, Saudi Arabia

**Keywords:** *Lysinibacillus*, insecticidal, larvicidal, remediation, bio-controlling agent

## Abstract

Microbes are ubiquitous in the biosphere, and their therapeutic and ecological potential is not much more explored and still needs to be explored more. The *bacilli* are a heterogeneous group of Gram-negative and Gram-positive bacteria. *Lysinibacillus* are dominantly found as motile, spore-forming, Gram-positive *bacilli* belonging to phylum *Firmicutes* and the family *Bacillaceae*. *Lysinibacillus* species initially came into light due to their insecticidal and larvicidal properties. *Bacillus thuringiensis*, a well-known insecticidal *Lysinibacillus*, can control many insect vectors, including a malarial vector and another, a *Plasmodium* vector that transmits infectious microbes in humans. Now its potential in the environment as a piece of green machinery for remediation of heavy metal is used. Moreover, some species of *Lysinibacillus* have antimicrobial potential due to the bacteriocin, peptide antibiotics, and other therapeutic molecules. Thus, this review will explore the biological disease control abilities, food preservative, therapeutic, plant growth-promoting, bioremediation, and entomopathogenic potentials of the genus *Lysinibacillus*.

## 1. Introduction

**Figure 1:**
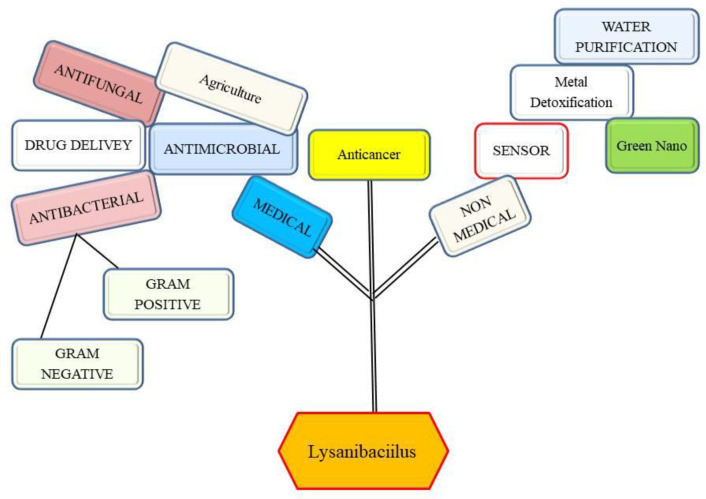
Potential applications of *Lysinibacillus* species in human welfare.

Bacterial isolates from different habitats have been reported to produce many valuable metabolites for human welfare. Numerous diseases have plagued humans from the beginning of time, and bacteria and fungi bring on the majority of these illnesses and are drug-resistant. Different therapeutic approaches have been used to treat diseases. Numerous investigations have been conducted since 1928 when a bacterium with antibacterial properties was discovered. Additionally, the utilization of microbial capabilities and the creation of antimicrobial substances against disease-causing factors. They have antimicrobial actions on bacteria, fungi, viruses, protozoa, and other etiological agents of human and plant illnesses [1]. Thus, many microbes, mainly bacteria like lactobacilli and gut microbiota including *Lysinibacillus* have been explored to serve human being a lot (Figure 1).

The level of living for people has significantly improved as a result of urbanization and industrialization. However, a significant amount of hazardous materials are produced due to various industrial advancements. Additional multivious contaminants are released into the soil, water, and air. For instance, mining operations are carried out to gather different metals employed in the metal industry. However, during processing, heavy metals such as Ni, Cu, and Zn leach into the environment [2]. Chemical fertilizers and insecticides are frequently applied in agricultural businesses to increase food output and satisfy worldwide food demand. The use of chemicals in food and agriculture indiscriminately degrades soil quality and pollutes the soil, water, and air [3].

Moreover, urbanization results in overcrowding, which creates ideal conditions for pathogens’ growth and diseases to spread, leading to several health issues. Insecticides frequently used to control insects and pests in agriculture are dangerous to the environment. The public’s worries regarding the long-term consequences of chemicals on the environment and human health have increased in recent years. Thus, reducing the usage of synthetic chemicals and hazardous metals, as well as chemicals, becomes a significant problem. Many microorganisms, bacteria, and fungi can be employed to lessen the harmful effects of chemicals produced by synthetic industrial processes [4]. The potentiality of microorganisms grows daily because of their benign and environmentally favorable character.

Numerous plant diseases as being susceptible to biocontrol by many members of the genus *Lysinibacillus*. A few of them, including *L. fusiformis* and *L. sphaericus*, have been described as bio-controlling solid bacterial strains, preventing, or treating various diseases of plants [5]. *Lysinibacillus* sp. synthesize a variety of antimicrobial substances that may have a role in the prevention or treatment of disease. It has been observed that numerous antibacterial chemicals and enzymes are produced by *Bacillus* sp. including *Lysinibacillus,* which can act as biocontrol for many diseases [6,7].

A bacteria known as *Bacillus thuringiensis* can control a variety of insect vectors that spread microbial diseases to people. Bacilli and a few other types of bacteria can clean up heavy metal-contaminated sources. Commercial products to increase crop yield based on *Pseudomonas, Bacillus*, and *Rhizobium* are now on the market as bio-controlling agents [5]. Various microorganisms, including Pseudomonas, Agrobacterium, Trichoderma, and many bacilli members, are used to create biocontrol products that effectively inhibit different plant disease types [8]. Because it produces endospores, highly resilient, specialized structures that can withstand various environmental conditions and facilitate commercial formulation, the genus Bacillus is among these bacteria that is typically used as the active ingredient in most microbe-based products due unique features of their spores. As a result, growers now trust products made with Bacillus. Researchers have been looking for promising agents to expand the repertoire of microbe-based products.

**Figure 2:**
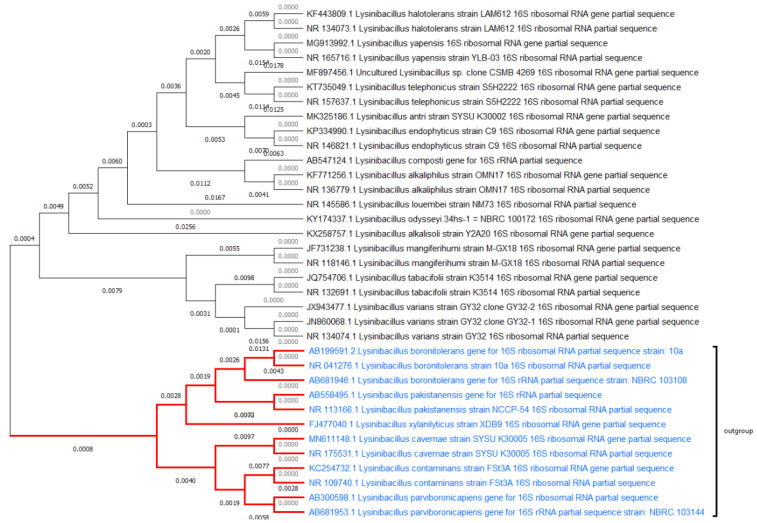
Phylogenic evaluation of *Lysinibacillus* species.

According to reports, *Lysinibacillus*, a newly created genus that was formerly categorized as Bacillus, can reduce insect populations, clean up habitats that have been contaminated with heavy metals, and boost crop yields. Since *Lysinibacillus* can create endospores can be regarded as an appropriate agent for microbial products [4]. The genus Lactobacillus is made up of a phenotypically diverse collection of rod-shaped, Gram-positive facultative anaerobic organisms [9]. Differentiation between the Lactobacillus genus members is possible. Biological, biochemical, and molecular traits allow for comparison with the other members. They used phenotypic characterization as a first step in describing various bacterial strains such as Lactobacilli and others. As a result, it is a general description—of physical, dietary, cultural, and biochemical characteristics [10]. The majority of these phenotypic tests are for culturable microorganisms. Hayat et al. (2013) describe the size, form, and other physical characteristics of the cell wall composition, cell surface properties, staining characteristics of spore production, and motility physical and nutritional [11]. It is crucial to have certain qualities [12]. Natural isolates’ phenotypes are used to characterize bacterial isolates. The *lysinibacillus* colonies were described as variables, including spherical and white or creamish off-white in appearance. Ryu Y et al. found that the spores made in different growing conditions have very other cell structures, which affects how long they live and how fast they grow [13]. Although they are distinct in habitat and physicochemical characteristics, they are very close to each other by evaluation (Figure 2).

Microbes are picky regarding the conditions of their culture, including the media, carbon and nitrogen sources, pH, temperature, and salt tolerance. As a result, these factors significantly impact how different species grow [6,7,14,15].

According to some phenotypic characteristics, some *Lysinibacillus* members have also been isolated from water, clinical samples, or food samples and described [16,17,18]. According to this, *Lysinibacillus* are widely found in various natural habitats and frequently grown in NB and LB. Moreover, MRS, a nitrogenous-rich medium supplemented with micronutrients like ammonium acetate, potassium hydrogen sulfate, tween 80, etc., favors the better growth of *Lysinibacillus* than the NB and LB. Hence, components might support the synthesis of enzymes or other proteinaceous components required for conducting life-supporting processes of the isolated bacterial cells. Moreover, the presence of ammonium acetate-like elements in MRS media might be involved in the neutralization of the acid, which was produced by the metabolic activities of the isolated bacterial cells. The maximum development pH of *Lysinibacillus* was reported at 6–10, but the highest growth was reported between pH 6 and 8. The *Lysinibacilli* were said to grow best between 30 and 37 degrees Celsius, moderately well between 20 and 40 degrees Celsius, and with the least amount of growth at 4 degrees Celsius, below 20 degrees Celsius, and above 40 degrees Celsius [6,7]. The slow rate of reaction, low solubility, and constrained diffusion of the nutritional components may be the causes of the nominal growth at low temperatures. Another crucial cultural trait that impacts organisms’ growth is the ability to tolerate salt—since sodium ions play a role in cellular osmolality and are essential for transporting amino acids. At 1–2 percent NaCl, it was reported that *Lysinibacillus* has grown at its best.

Based on their physiology, distinct peptidoglycan composition, and evolutional tree position based on 16 S rRNA gene sequences, *Bacillus fusiformis*, and *Bacillus sphaericus* were reassigned to the new genus *Lysinibacillus* [6,7]. The presence of aspartic acid characterizes the cell wall peptidoglycan, known as Lysine A4a (Lys-Asp) type.

## 2. Insecticidal Potential of *Lysinibacillus*

Chemical pesticides are the most common way to get rid of insect pests that can spread serious diseases to humans and animals being toxic. Toxins are being misused or overused due to increased agricultural products, necessitating more harmful effects and more significant concentrations of insecticides. As a result, toxic insecticide levels in the bodies of endpoint consumers such as a human and predators have increased, as have the adverse effects cohabit environment and agroecosystem. Ecologically speaking, bioinsecticides are preferred over synthetic insecticides because they must be poisonous to the target organism while being minimally or completely non-toxic to non-target animals and humans [19]. The strong insecticidal potential of many plant products has been demonstrated in testing. The *Lamiaceae Diterpenes* have reported promising results in the quest for plant chemicals with bio-insecticidal action [19]. P-menthane-3,8 diol, from members of the family *Annonaceae*, chemical components of neem (*Meliaceae*) against *An. gambiae Ae. aegypti Ae. albopictus An. stephensi Cx. Quinquefasciatus, Rotenone, Flavonoids* against *Ae. Aegypti* have been reported with larval activity [19,20].

Researchers have recently begun to admire microbial pesticides because they are safe and environmentally benign. The active components of microbial pesticides include a variety of entomopathogenic microorganisms. Direct application of a disintegrating bacterium product solves the pesticide residue problem in crop output and allows for the manufacture of safe, pollution-free farm commodities. A novel fluoroglycofen-degrading Gram-positive *Lysinibacillus* Strain cmg86 (T) was recovered from herbicide-polluted soil of Tongjing, Jiangsu province, China [21].

It has been well-reported that *L. sphaericus* has entomopathogenic properties [22]. In 1965, this bacteria was initially identified as a mosquito parasite [23]. After that, a complete isolation and screening program was set by World Health Organization. Substantial larvicidal activities were reported from an Indonesian isolate of *L. sphaericus* strain 1593. They attracted researchers’ attention because a bacterium may be able to suppress the malaria vector mosquito, and soon it was screened and commercialized by many pharmaceuticals such as Bthek Ltd., Brazil, Santa Maria, and commercializing Sphaerus SC. Product Valent Biosciences, Illinois, USA; VextoLex FG, Habana, LABIOFAM, Cuba; GRISELESF^®^ Novo Nordisk, Bagsvaerd, Denmark trading as Spherimos. The Culex quinquefasciatus has been effectively managed to reduce not only the population dramatically but also infected bites were down by almost 60% by the application of insecticidal Spherimos. Vectolex CG^®^, a commercially available product of *L. sphaericus*, effectively controlled malaria-causing *Anopheles gambiae sensu lato,* and Saint Louis encephalitis-causing mosquitoes *Culex pipiens, Culex restuans*, and *Aedes triseriatus* [24,25]. The *Lysinibacillus* cells produce a binary toxin that kills cockroaches and caterpillars while Cry, and Mtx toxin produced by the Bacillus is mosquitocidal. In addition to its effect on mosquitoes, *L. sphaericus* also kills other insect pests, like common cutworm (*Spodoptera litura)*, German cockroach (*Blattella germanica*), and nematodes [26,27], suggesting that this *Lysinibacillus* may be effective for eradicating a variety of animal pests, including insects. Numerous insect-killing toxins, including mosquitocidal toxins, sphaericolysin, S-layer protein, Cry48/Cry49 toxin, and binary (Bin) toxin, are produced by Entomopathogenic *L. sphaericus* [24].

The full pathogenicity of Cry48Aa toward mosquito larvae and its combination with Cry49Aa as a binary toxin is attributed mainly to its N-terminal domain [28]. Bin toxin, produced during the late sporulation phase, is responsible for its killing effect on insects. *L. sphaericus* strains that produce bin toxin have a potent insecticidal effect on mosquitoes. Recombinant DNA technology has been used successfully to create a hybrid system for the production of desirables [27]. The *Bacillus sphaericus* larvicidal protein Mtx1 and Mtx2 toxins co-expressed in the bacterial system using *Escherichia coli*, and *A. aegypti* larvae were effectively killed through the synergistic action of both toxin Mtx1 and Mtx2 toxins co-expressed in *Escherichia coli* [29].

Recently bin toxin was described as an anticancer and drug cancer delivery system in cancer future hope for drug delivery targeting mitochondria. In the liver cell line HepG2, treated with bin toxin a and b (50 g/mL), the intracellular location of these toxins was described with altered cellular morphological features with decreased cell survival. In tests on Hs68 cells, bin toxin exhibited no toxicity. In hepatic cells, 24 ng/mL for PS2 and 46.56 and 39.72 g/mL for both A and B toxins were observed, respectively, in hepatic cells. Increased caspase levels in experimental cell lines proved that apoptosis had been induced [30]. Moreover, the anticancer effects of toxins from the *Bacillus thuringiensis* were potentially observed with cancer cells like Caco-2, A549, HepG2, KKU-M055, and HK-1, in which HK-1 is most susceptible to toxins [21,31,32].

High synergism was found against *Culex quinquefasciatus* and *Aedes aegypti* larvae when *Bacillus thuringiensis* protein Cyt2Aa2 and Cry4Bawere co-expressed in *Escherichia coli*. Recently, Chutchanun Trakulnaleamsai et al. created a recombinant strain of *Bacillus subtilis* and successfully described the production of *Lysinibacillus sphaericus* Mosquitocidal Protein Mtx2. The expressed protein has been reported to display synergistic action of other insecticidal proteins and could be used in combination to control the insect more significantly [33]. One of the active molecules most frequently applied to manage perennial weeds worldwide is herbicides with a glyphosate base. This substance is particularly resistant to biodegradation and has the propensity to enter aquatic environments that might harm unintended species like mosquito larvae. Numerous arboviruses, including Zika, and dengue, are spread by mosquitoes, *Aedes aegypti, L. sphaericus* 2362, and IIIare spore-forming bacterium that can also kill *Ae. aegypti* larvae can break down glyphosate into non-toxic forms of chemicals. Moreover, *L. sphaericus* 2362 and III with glyphosate has synergistic killing effects on Aedes aegypti larvae [34].

Cypermethrin was highly susceptible to degradation by *L. cresolivuorans* HIS7, with time-dependent degradation rates, which was reported to increase from 54.7 to 93.1 percent after 7 to 42 days, respectively. In the maize crop, the qualitative study described that the rate at which bacteria broke down cypermethrin in the soil changed over time [35].

## 3. Antibacterial and Antifungal Potential of *Lysinibacillus*

The bacterial members are renowned for producing organic acids, alcohol, antibiotics, phytohormones, and lytic enzymes to boost growth, reproduction, and yield. *Lysinibacillus odysseyi* KC149512 was replated with antimicrobial potential. According to the mass spectrometry and gas chromatography-based characterized fraction, the antimicrobial activity is due to furan, butenyl methyl ketone, and diazene [36]. Lysinibacillus sphearicus strain PRE16 has been reported to inhibit the growth of *Staphylococcus aureus*, *Escherichia coli*, and *Bacillus subtilis* by 18.00 ± 2.00, 20.00 ± 4.00, and 23.00 ± 2.00, respectively [37]. A member of *Lysinibacillus* was reported to produce an antibacterial protein of nearly 51 KDa, which has been shown to have an inhibitory effect on foodborne pathogens after being isolated from rotten fruit and vegetable wastes. Apart from the antibacterial effect, the two other members of Lysinibacilli have been reported to have antifungal effects against *Aspergillus* spp. and *Fusarium* [6,7,38]. The connection between seaweed epiphytic bacteria and their hosts is poorly understood. Algal variety is abundant in the Andaman Islands’ marine environment, but little is known about how these organisms interact with microbial populations. *Lysinibacillus* sp. strain AS_1 reported broad-spectrum inhibitory activity against the pathogenic bacteria with MIC values ranging from 4 to 8 mg/mL. Cell-free supernatant of *Lysinibacillus* sp. *strain AS*_1 and other isolates such as *Peribacillus* sp. strain AS_2 and Bacillus sp. strain AS_3 were described with growth inhibition of more than 90% against all the cancer cell lines at a concentration of 1000 g/mL [39]. Researchers have already looked into how well a peptide-gene-linked product from *Lysinibacillus spharicus* kills bacteria. In one study, *Lysanibacillus*, which has the potential to combat bacterial wilt disease in chili, demonstrated significant biocontrol efficacy (88.9% and 66.7%). Bacilysin, surfactin, and fengycin genes are well expressed for antimicrobial activity, and PCR amplifications have confirmed their presence.

The antifungal potentialities of *lysanibacillus* against *Colletotrichum acutatum*, *Aspergillus niger, Fusarium moniliforme, Fusarium oxysporum*, and *Fusarium solani* have been explored by many previously described studies (Table 1). It is believed that the strong inhibitory effect of *Lysinibacillus* is due to the production of volatile organic molecules, including unsaturated hydrocarbons, aromatic hydrocarbons, alcohols, aldehydes, acids, and ketones [6,7,38,40].

A diazotrophic endophyte, *Lysinibacillus sphaericus*, from rice has been reported effective against the rice sheath blight pathogen, *Rhizoctonia solani*. The application of *L. sphaericus,* either alone or in combination, induces systemic resistance, as evidenced by the considerable buildup of defense enzymes such as polyphenol oxides and peroxides, phenylalanine ammonia, and phenolic compounds [41]. A novel isolated bacterial strain demonstrated a strong inhibitory effect on fungi such as aflatoxigenic *Aspergillus parasiticus* and *Aspergillus flavus*. It was reported that *Lysinibacillus* isolates can produce and act as a protease over a broader range of temperatures and pH levels, as well as synthesize a strong inhibitory effect on pathogenic fungi [66]. Chitinase, a biocontrol agent against plant pathogenic fungi, is a product that chitinolytic bacteria can create. *Brevibacillus reuszeri* and *Lysinibacillus* were found to be antifungals against *R. solani* and *F. oxysporum*. Both bacteria were able to stop the growth of the fungus, as shown by the activity of *L. fusiformis* (30%) and *B. reuszeri* (77%) [67].

A member of the Allylamine class, “Terbinafine,” that kills fungi recovered from *Lysinibacillus* Isolate MK212927 was recently described. The isolated bioactive molecule kills fungi and maintains its antifungal effect up to 60 °C, while optimum fungicidal activity was observed with a narrow pH range (6–7.8); moreover, the biomolecule did not lose its activity in the presence of several surfactants and enzyme [68]. *Lysinibacillus* fusiformis strain S4C11 has antifungal activity against various fungal infections and can prevent Botrytis cinerea conidia from germinating and impede its growth by producing volatile organic compounds [42]. The antifungal metabolites 1,2-benzenedicarboxylic acid butyl,2-ethylhexyl ester, a strong antifungal volatile organic compound, has been recovered and tested. Among rice’s most common crop infections, sheath blight is caused by *Rhizoctonia solani*. It is a significant barrier to the cultivation of rice in not only India but also the majority of rice-growing nations in Asia [41]. Moreover, the effect of organic volatile compounds recovered from *Bacillus velezensis* CT32 on Fungi *Verticillium dahliae* and *Fusarium oxysporum* has been identified [69].

## 4. Metal Remediation Potential of *Lysinibacillus*

Modernization resulted in high human activity over the previous few years, and varied environmental contamination has become an issue for the globe [70]. The primary ecological concerns we currently face include water, soil, and air contaminations from fuel, aromatic hydrocarbons, heavy metals, synthetic substances, and industrial waste. Both industrial agriculture sectors use metals to make alloys, insecticides, and electrical and electronic goods. Additionally, the environment is frequently contaminated by hazardous metals released from industrial effluents, including petroleum, agriculture, pharmaceuticals, and mining operations. Physical and chemical processes such as precipitation, electrochemical, ion exchange, and membrane techniques are frequently employed to treat hazardous waste. However, these processes can sometimes not be put into place well because of technological or financial problems [71,72]. Thus, a cost-effective process catalyzed by microbes has been described effectively. Several species, including *L. fusiformis, L. sphaericus,* and *L. macrolides* have been related to have remediation potential. *Lysinibacillus* sp. is one of the bacteria described by Kamaruzzaman et al., isolate 10 J, and is resistant to lead at concentrations up to 300 mg/L [43]. The findings demonstrated that highly lead-resistant rhizobacteria have the strength to acquire features that promote plant growth and have the capacity to support the development and productivity of *S. grossus*.

The bacteria associated with enhancing the growth of plants have been reported to promote the growth of plants under stressful environments through mineral solubilization, siderophore, phytohormones, 1-aminocyclopropane-1-carboxylic acid (ACC) deaminase, and other things. It has been observed that several bacterial members, including *Lysinibacillus, Bacillus*, Pseudomonas, *Acinetobacter, Serratia, and Gluconacetobacter*, can solubilize and resist Zn [73].

Enhanced crop biomass was reported by *Lysinibacillus* strains (SKTS17 and SKTS20) in maize seedlings. This made the plants grow faster and take in more zinc when stressed by zinc (SKTS20 and SKTS26) [74].

Along with the qualities listed above, metal-tolerant bacteria can release a range of metabolites essential for mobilizing toxic substances in flora. A growing body of evidence indicates that in addition to the traditionally recognized magnesium level, Mn exposure results in toxicities in many environmental settings, such as contaminated foods, food sources, infant formulas, soil, air, and water that natural or artificial agents have polluted. Neurological toxicities are commonly observed in Mn-exposed areas. Microbes, bacteria, and fungi play a significant role in the cleanup of the environment. A *Lysinibacillus* sp. remediated the Mn from mine waste through the bioleaching process. After 21-day research under optimal circumstances of pulp density 2% (150-m particle size), temperature 30 °C, and pH 6.5, This species of *Lysinibacillus* has been reported to catalyze the Mn bio solubilization with 84% efficiency [75,76]. The PGP characteristics of the *Lysinibacillus* species, potassium, were also solubilized with the formation of siderophore and indole acetic acid (60.0–84.0 g/mL).

Additionally, the *Lysinibacillus* members were tested in *Zea mays* under greenhouse conditions with and without a 2 g kg^−1^ Zn stress. The findings demonstrated that in comparison to control crops, *Lysinibacillus* sp. treated seeds improved plant characteristics growth and biomass yield in both situations. Under Zn-toxic soils, the increase in root development ranged from 49.2 to 148.6%, and the promotion in shoot length from 83.3 to 111.7%. The culture-treated seedlings significantly increased their proline levels, ascorbic acid, total phenol, and chlorophyll a and b. Compared to control plants, maize roots’ Zn intake ranged from 31.5 to 210.0 percent. As a result, this study suggested that Z. mays may be grown in agricultural fields exposed to Zn using the tested Zn-tolerant *Lysinibacillus* species [74].

A decline in crop output is brought on by the metal pollutants of crop fields, particularly with trace metals such as Mn, Al, Cu, Zn, and Pb. When more metals are in the soil, many *Lysinibacillus* species can help plants grow. For instance, inoculum treatment of soil using Pb-tolerant *L. sphaericus* strains dramatically increased the development of jack beans. There have also been reports of Zn-resistant *Lysinibacillus* sp. having a growth-association impact on maize crops in Zn-polluted soil [74,77].

The strain of *L. fusiformis* can tolerate mercury, and this could be due to the enzymatic reduction conversion of hazardous HgCl_2_ to HgCl. Moreover, the reduction process has also been reported with *L. sphaericus* in detoxifying Hg and Cr. An endophytic, *Lysinibacillus* fusiformis, synthesizes acetic acid, coordinates jasmonate signaling, and helps prevent cadmium uptake in tomato plants [78].

The bioremediation of Cr(VI) through a novel *Bacillus* sp. CRB-B1 strain has been described by biosorption and Bio reduction processes. Compared to cell-free samples and debris, the supernatant without cells had a high Cr(VI) remediation percentage of 68.5 percent. This implies that the external reductase enzyme could be a possible mediator of Cr(VI) reduction, which occurs primarily extracellularly. Only a minor quantity of the decreased Cr was retained in the cells; most of it was disseminated in the surrounding culture medium [79,80].

*L. macrolides* and *L. xylanilyticus* have been reported to detoxify toxic Se in elemental Se nanoparticles [74]. Chromium (Cr) reduction strains (*Bacillus* sp. AK-1 and *Lysinibacillus* sp. AK-5) were utilized to treat Cr-contaminated soil [81].

*Lysinibacillus xylanilyticus* and *Lysinibacillus macrolides* can potentially reduce selenium extracellularly or intracellularly.

A novel *bacillus* known as *Lysinibacillus* strain HST-98 can effectively reduce Cr(VI), even at a high concentration of 250 mg/L, through intracellular enzymes. The optimum conditions to minimize the metal were pH 8–9, at 36 °C, in the presence of sodium lactate as an electron donor. While Ni^2+^, Cd^2+^, and Zn^2+^ are detrimental to Cr(VI) reduction, coexisting metal ions such as Co^2+^, Mn^2+^, and Cu^2+^ have been reported as beneficial to isolated strains [82]. Transcriptome analysis of a *Lysinibacillus* strain 15-4 reported that eight important KEGG and 1098 DEGs pathways strongly interacted with Cr(VI). The essential processes involved in the survival of *Lysinibacillus* in response to Cr(VI) were translation and porphyrin sulfur metabolism [83].

Archaea and bacteria use cellular proteins as a protective layer against the extracellular environment at their bio-interfaces. The variable pH values (2.0–9.0) were used to describe the S layer protein interaction with metal conc. Cm (III) (0.88 M). Specific and non-specific binding sites may be differentiated using the ensuing luminescence spectra and lifetime data. A notable emission band with a strong peak at 602.5 nm and a long lifetime of 310 s was observed, confirming the particular Cm (III) binding to S-layer proteins [84].

Typically, *Lysinibacillus* undergoes reduction and biosorption mechanisms during the remediation operation. Through the reduction, poisonous metals are transformed into non-toxic forms through the enzymatic machinery of the microbial cells. The surface protein layer (paracrystalline S-layer) and other structural function groups of the *Lysinibacillus* sp. cell walls, including the carboxyl, carbonyl, hydroxyl, amide, imidazole, phosphodiester, and phosphate, such as ionic groups, are essential for metal sorption by the *Lysinibacillus* cellular system. It was believed that electrostatic attraction operated between ionic groups on microbial cell walls and metal ions, resulting in their engulfment by the cell, detoxifying them by a reduction process [85,86,87].

The *Lysinibacillus* sp. was trapped in the holes of porous ceramic. The immobilized porous ceramsite (IM-PC) was supplemented with 1.5:10 (g/g) for a more prolonged release of nutrients to provide *Lysinibacillus* sp. JLT12 with low secondary pollution [78].

Arsenic-treated worms fed *L. sphaericus* lived much longer than those *E. coli* and expressed more of the host defense, stress tolerance, and antioxidant enzyme sensitivity genes (cyp-35A2, isp-1, ctl-2, and sod-1) while accumulating fewer reactive oxygen species (ROS). The *L. sphaericus* B1CDA diet boosted C. elegans fitness in contrast to *E. coli* while detoxifying arsenic-induced ROS and lengthening longevity [88].

The examined strains of *L. sphaericus* have the sorption and manufacturing capabilities that make them a suitable replacement for biomining procedures, particularly those involving gold extraction [89].

*Lysinibacillus sphaericus* CBAM5 can remove gold metals immobilized on alginate microcapsules and polycaprolactone (PCL) micro-fibrous mats by accumulation and adsorption. Thus, these materials and bacterial species could be employed to accumulate gold from industrial wastewater [90].

**Figure 3:**
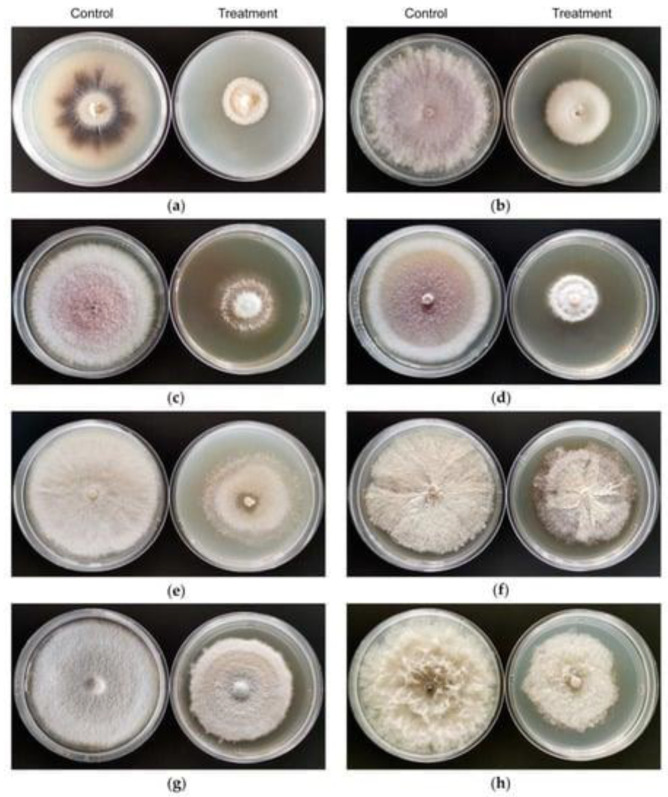
Effect of VOCs emitted by strain CT32 on the growth of 8 phytopathogenic fungi in vitro. In the sealed plates test, fungi in the control groups were cultured on potato dextrose agar (PDA) medium. The mycelial growth of fungi in the treatment groups was suppressed upon exposure to volatiles emitted by strain CT32. (**a**) *V. dahliae*; (**b**) *F. oxysporum* f. sp. *fragariae*; (**c**) *F. oxysporum*, f. sp. *niveum*; (**d**) *F. oxysporum*, f. sp. *cucumerinum*; (**e**) *B. cinerea*; (**f**) *T. cucumeris*; (**g**) *G. cingulata*; (**h**) *B. dothidea*. Adapted from Li et al., 2020 [69].

**Figure 4:**
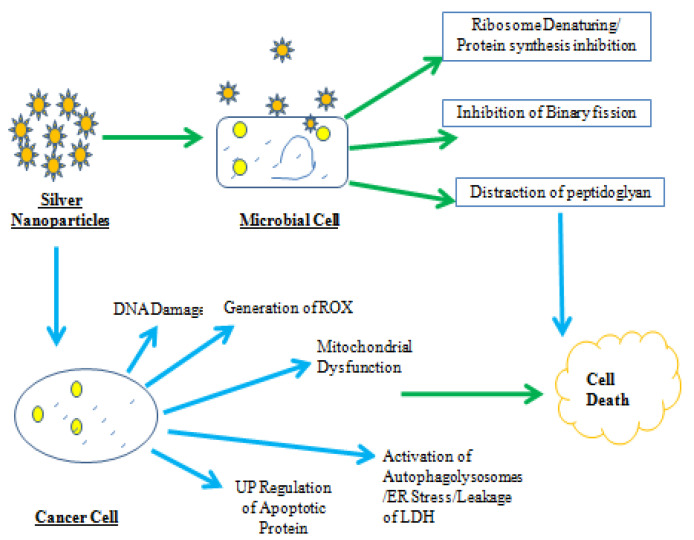
The silver nanoparticles synthesized by *Lysinibacillus* sp. and their potential possible applications.

Moreover, AgNPs produced by *L. xylanilyticus* MAHUQ-40 served as potent antibacterial agents that inhibit bacterial pathogens. The potentials of synthesized silver nanoparticles and their applications are shown in Figure 3 and Figure 4.

## 5. Plant Protection as Well as Plant Growth Promoter Potential of *Lysinibacillus* against Bacteria and Fungi

It is always desirable to search for an ideal way that helps to increase crop output in an environmentally responsible manner; plant growth helping microbes, mainly bacteria, has attracted particular interest from the scientific and agricultural communities.

It is always good to look for the best way to increase crop yield in a way that is good for the environment. Plant growth-helping microbes, mainly bacteria, have attracted particular interest from the scientific and agricultural communities. The ability of specific bacteria found in plants to promote plant development is widely documented. Plant growth-promoting rhizobacteria (PGPR) and plant growth-promoting bacteria (PGPB) are common names for these helpful bacteria. *Rhizobium, Azotobacter, Pseudomonas, Azospirillum*, and *Bacillus* species are previously well-known examples. It has been well reported that these microbes promote growth through the fixation of nitrogen, solubilization of minerals, the generation of phytohormones, and others PGPB and PGPR can directly and indirectly boost plant growth development [44,91].

Nitrogen fixation is essential for the cultivation and productivity of a crop, and in recent years, crop rotation has been used by farmers. Many Korean crop plants, such as sesame, rice, wheat, soybeans, pepper, and lettuce, were reported to contain free-living nitrogen-fixing bacteria with significant nitrogenase and indole acetic acid activities, including *Bacillus fusiformis* [45]. PGPR-*Lysinibacillus fusiformis* B-CM18, an kitinase enzyme-synthesizing bacterium, was found in the rhizosphere of chickpeas. It was discovered to synthesize multiple PGPR activities and have in vitro antifungal activity against various fungal plant diseases [46]. *Rhizobacteria*, which help plants grow, are being used more often these days as an excellent alternative to chemical fertilizers because they can be used as biofertilizers. Islam et al. reported that *Lysinibacillus* also shows nitrogen-fixing potential other than the *Ochrobactrum* sp., *Novosphingobium* sp., *Brevundimonas* sp., and *Sphingomonas* sp., on the growth promotion of red pepper and tomato [47].

According to current research, *Lysinibacillus* species, including *L. xylanilyticus*, *L. fusiformis*, *L. sphaericus,* and *L. chungkukjangi*, have various advantages and characteristics aid in promoting plant growth. For the development of plants and their product production, nitrogen is the most restricting main element, such as P and K. Although the quantity of accessible N in the rhizosphere is constrained, plants can still take N mainly from the soil through their rooting system. Despite making up a high amount of nitrogen (80%) in the earth’s atmosphere, plants cannot utilize nitrogen gas [92]. The *L. sphaericus* strains isolated and characterized were examined for their ability to fix nitrogen, nitrate, and generate indoleacetic acid. The nitrification of nitrate (0.26–0.7 mg/L), fixing of nitrogen (0.61–1.47 mg/L), and the production of IAA (3.3–5.5 g/mL in L-tryptophan media and 0.3–1.1 g/mL in trypticase broth) were all accomplished by *L. sphaericus strains* [48]. The maximum phosphate dissolution rate and IAA acid generation efficiency were demonstrated by *Lysinibacillus* sp. *S24*, which produced 135 05 g ml1 [49].

*Lysinibacillus* (EPR2) exhibits tremendous promise for reducing *F. verticillioides* caused by maize illnesses, enhancing seedlings, and the nutritional value of maize [50]. In field testing and greenhouse trial, it has been demonstrated that the bacterial strains *Lysinibacillus sphaericus T-19*, *Paenibacillus alvei T-22*, and *Paenibacillus alvei T29* are efficient plant growth promoters and biocontrol agents of plant disease [51]. Yadav et al. isolated and analyzed the plant growth-promoting activities of 395 isolates, including *Lysinibacillus*, *Exiguobacterium, Paenibacillus, Planomicrobium, Planococcus, Staphylococcus*, and *Sporosarcina*. These bacteria promote direct plant growth through the solubilization of potassium, phosphorus, and zinc, as well as indirect growth through various means such as phytohormones, nitrogen fixation, and 1-aminocyclopropane-1-carboxylate deaminase activity. Moreover, they also evaluated the antagonistic production of siderophore lytic enzymes, ammonia, and hydrogen cyanide [93]. In Ain Defla (Northern Algeria), *L. fusiformis strain Lf89* was first described in the rhizosphere of the tomato plant and signified the growth-associated activity on the tomato plant [56]. An endophytic bacterium of *Lysanibacillus* sp. has reported a growth promotion effect, an increase in the chlorophyll contents of plants, and an enhancement of antioxidant enzymes [57]. One of the primary obstacles to the global development of vegetables is root-knot nematodes (RKNs). Utilizing the protoplast method, RKN Meloidogyne incognita was combated by the *Lysinibacillus sphaericus Amira* and *Plantarum SA5* strains. In the bioassay and greenhouse testing, their fumigants were evaluated for their nematocidal and chitinase activity [58].

Phosphorus is needed in several cellular processes. It has been shown that several *Lysinibacillus* species can convert fixed organic forms of phosphorus into easily soluble P forms that are simple for plants to absorb. Moreover, several *Lysinibacillus* strains can dissolve other significant hydrophobic forms of minerals, including K, Fe, Zn, and silicate [52,53].

As a waste product from the phosphoric acid manufacturing process in the fertilizer business, phosphogypsum (CaSO_4_) is created. Only 15% of the world’s supply of phosphogypsum is recycled, and the remaining 85% is stored in massive piles close to companies, creating risks to the environment and human health. Sulfate-reducing bacteria (SRBs) have been extensively studied [54,55]. Naureen et al. 2017 investigated their ability to biotransform phosphogypsum into calcium carbonate or calcite (CaCO_3_), which is a lengthy process that produces the dangerous gas H2S gas. *Lysinibacillus sphaericus* strain GUMP2 strain could effectively transform phosphogypsum into agricultural fertilizer, crystalline, bead-shaped CaCO_3_, and ammonium sulfate [94].

This *Lysinibacillus* sp. make it easier to transform insoluble minerals into bioavailable forms by secreting organic acids, hydrolytic enzymes, and metal chelator compounds. Many researchers indicated that *Lysinibacillus* culture treatment significantly improved crop growth and output. According to a recent study by [59], maize plants’ shoot growth was greatly enhanced when the soil was soaked in pumpkin rhizosphere. The isolate, *L. sphaericus*, catalysis phosphate solubilization for improved crop growth. Wheat plants and soybeans had their shoot and root biomass boosted by IAA producers *L. chungkukjangi* and *L. fusiformis* culture treatments to the root of the crop. The enhanced shoot growth of many crops such as hot peppers, sweet peppers, chicory, leeks, and green beans has been observed with root supplemented with *L. fusiformis* culture, producing siderophore and IAA for better shot growth and increased biomass of fruit zucchini [60].

Plant development and various abiotic and biotic stress tolerance are significantly influenced by plant hormones [95]. Plant growth promotion by growth-associated bacteria is linked to the capacity of microbes to produce cytokinin, indole-3-acetic acid (IAA), and gibberellin [96,97,98]. Many *Lysinibacillus* species have been reported to produce these hormones [57,98]. Abiotic stress has a long-lasting detrimental effect on the growth of plants and causes a significant loss in crop yield. Additionally, *Lysinibacillus* species have been shown to boost the growth of plants under unfavorable stress circumstances and increase plants’ resistance to various abiotic stresses [61,62,64]. One main issue affecting global agricultural output is saltiness [99]. Under salty conditions, putting salt-resistant bacteria in the soil of a crop could be an excellent way to help crops grow and produce more. The survival of roots and shoots under saline conditions was significantly improved by the root of the crop treated with a salt-resistant *Lysinibacillus* species. Phytopathogenic viruses are potentially inhibited by *Lysinibacillus* fusiformis strain S4C11.

The perfect mechanisms of *Lysinibacillus* that promote plant growth have not been thoroughly explored. It has been described that accumulation of ascorbic acid, phenol, and proline-like potential reducing agent, as well as superoxide dismutase, peroxidase, and catalase enzymes systems in the plant, may be crucial in improving plant growth under various stress conditions [59,65]. *Lysinibacillus* sp.M4 has been described as forming a biofilm on plastic surfaces, and this bacteria could be used to degrade plastic [100].

Formaldehyde is produced from methanol by the action of methanol dehydrogenase (Mdh). *Lysinibacillus xylanilyticus* (Lxmdh) has produced methanol dehydrogenase, which could benefit the bioremediation of organic alcohol [101,102].

Numerous bacteria secrete biosurfactants, which are amphipathic bio-molecules with significant surface activity. In order to evaluate the output and surface functional capabilities of *Lysinibacillus* fusiformis MK559526, which was isolated from soil in an auto repair shop, biosurfactant synthesis was studied.

The surfactant properties described with this strain were an emulsion index of 65.15 0.35%, oil displacement of 0.26 mm, hemolysis of 7.7 mm, positive drop collapse test found for the *Lysinibacillus* fusiformis surfactant. The optimum culture conditions for the synthesis of biosurfactants were 35 °C, 7.0 pH, 40 g/L of starch solution, and 1.5 g/L of urea. These circumstances resulted in a decrease in surface tension to 28.46 1.11 mN/m [103]. *Lysinibacillus* sp. shows excellent promise for producing PHB on an industrial scale by employing SCB as a low-cost substrate [104].

*Lysinibacillus* macrolides have been described with probiotics properties that can enhance the immune properties [100].

Due to its extraordinary benefits, the use of naturally derived adsorbents for bioremediation of dye-like toxins is becoming increasingly important—microscopic examination of extracellular polymeric material (EPS) produced by *Lysinibacillus* sp. SS1 reported a porous and rough surface. Within 30 min, almost 82 percent of the MG (100 mg/L) was adsorbed onto 2.5 mg EPS [63,105]. *L. sphaericus* strains were able to fix nitrify (0.26–0.7 mg/L of nitrate), nitrogen (0.61–1.47 mg/L of ammonium), and produce IAA (3.3–5.5 µg/mL in L-tryptophan media and 0.3–1.1 µg/mL in trypticase broth [63]. Thus, the EPS of *Lysinibacillus* or this strain could be an excellent source of water decolorizing the contaminated resources. The metal tolerance *Lysinibacillus,* boron tolerant, has been reported with antibacterial properties, and metal tolerance species of *Lysinibacillus* has also been reported to synthesize the antibacterial activity against Gram-positive and Gram-negative bacteria. This study highlighted the significance of microbially generated L-rGO in biofilm development and showed its potential for application as a doped polymeric in tissue engineering scaffolds [63,106].

## 6. Conclusions

The abundance of microorganisms in nature can be used to screen for competitive organisms that will help control human diseases and encourage crop production. Most unfavorable conditions, such as extremes of alkalinity, acidity, temperature, water, nutrient deficiency, and salinity, have been solved and characterized for significant applications in agriculture, medicine, industry, and environments. Bacterial communities are linked with plant growth and development in these conditions. Within the confines of accepted norms, the use of *Lysinibacillus* bacteria to manage dangerous infections may be a promising alternative strategy that may soon prove to be an invaluable tool for future attempts in a variety of disciplines. Instead of lessening the burden of clinical infections, *Lysinibacillus macrolides* worked as a biocontrol agent by regulating different pathogens in plants and vegetables. Bacterial species that can live inside the tissues of plants as endophytes and colonize the phyllosphere and rhizosphere are linked to plant growth. In addition to producing the phytohormones indoleacetic acid and gibberellic acid, solubilizing zinc, potassium, and phosphorus, and binding minerals, the microbiomes connected to crops can also trigger plant defense responses against pathogens and support plant growth in challenging conditions. Rapid industrialization has boosted economic growth and raised our standard of living. However, significant environmental contaminants have also been introduced into the atmosphere, causing various issues, including ecological disruption, environmental deterioration, and health effects. The challenge is to get the positive effects of industrialization and urbanization while reducing their adverse effects is the one we face. The *Lysinibacillus*-based techniques are used to reduce the number of contaminants produced by human activity and toxic compounds that accumulate in the atmosphere of water, air, and soil. One of the automated approaches to natural habitats is the use of these beneficial strains of *Lysinibacillus* bacteria found in natural ecosystems. Thus, such microbes might reduce their potentially harmful effects by lowering the use and toxicity of artificial chemicals. *Lysinibacillus* was once thought to be a good source of antibacterial, antifungal, and biopesticides, as well as bio stimulants for plant or crop growth and bioremediation. So, more research needs to be done to find new strains of *Lysinibacillus*, their active metabolites, and how *Lysinibacillus* can be used.

## Figures and Tables

**Table 1:** *Lysinibacillus* potentialities and their bioactive molecules.

Species	Origin	Active against	Possible Mechanism	References
*L. sphaericus*	Rice	Rice sheath blight (*Rhizoctonia solani*)	Induction of systemic resistance, production of antifungal volatile compounds, biosurfactant, HCN, and siderophore	Shabanamol et al., 2017 [41]
*L. sphaericus*	Rice	*Rhizoctonia solani*	Mycolytic activity	Shabanamol et al., 2018 [41]
*L. sphaericus*	Rice		Nitrogenase activity, IAA production, GA production, CK production, ACC deaminase production	Shabanamolet al., 2018 [41]
*L. fusiformis*	Apple	*Aspergillus nigri*, *Botrytis cinerea*, *Phomopsis viticola*, and *Rhizoctonia solani*	Production of antifungal compounds	Passera et al., 2020 [42]
*Lysinibacillus* sp.	Giant bulrush		Pb tolerance, NH3production, IAA production	Kamaruzzaman et al., 2020 [43]
*L. sphaericus*	Spinach		P solubilization, IAA production, NH3production	Sharma and Shaharan, 2015 [44]
*L. fusiformis*	Soybean, rice		Nitrogenase activity, IAA production	Park et al., 2005 [45]
*L. fusiformis*	Chickpea	*Fusarium oxysporum, Fusarium solani*, and *Macrophomina phaseolina*	Production of chitinase, protease, and β-endoglucanase	Singhet al., 2013 [46]
*Lysinibacillus* sp.	Rice		IAA production, ACC deaminase production, SA production	Islam et al., 2013 [47]
*L. sphaericus*	Culture collection		N fixation, nitrification, IAA production	Martínez and Dussán, 2018 [48]
*Lysinibacillus*	Tea		P solubilization, IAA production, siderophore production, ACC deaminase production	Borah et al., 2019 [49]
*Lysinibacillus* sp.	Maize	*Fusarium verticillioides*	Chitinase production	Abiala et al., 2015 [50]
*Lysinibacillus* sp.	Maize		P solubilization, IAA production	Abialaet al., 2015 [50]
*L. sphaericus*	Undescribed		N fixation	Labuschagne et al., 2015 [51]
*L. xylanilyticus*	Wheat		NH3production, ACC deaminase production, Zn solubilization	Verma et al., 2014 [52]
*L. sphaericus*	Wheat		N fixation, IAA production, P solubilization, K solubilization, Zn solubilization	Verma et al., 2016 [52]
*L. fusiformis*	Ginseng		P solubilization, IAA production, siderophore production	Vendanet al., 2010 [53]
*L. fusiformis*	Citrus	*Candidatus liberibacterasiaticus*	Production of salicylic acid and chitinase	Trivediet al., 2011 [54]
*L. sphaericus*	Maize	*Alternaria alternata*, *Curvularia lunata*, *Aspergillus* sp., *Sclerotinia* sp., *Bipolaris spicifera*, and *Trichophyton* sp.	Production of antifungal metabolites, hydrolytic enzymes, and siderophore	Naureen et al., 2017 [55]
*L. sphaericus*	Maize		IAA production, siderophore production, P solubilization, K solubilization, Si solubilization	Naureen et al., 2017 [55]
*L. fusiformis*	Tomato		No data	Rahmoune et al., 2017 [56]
*Lysinibacillus* sp.	Compost	Endophytic bacteria Growth promotion under salt stress conditions	Increase in chlorophyll contents of plants, enhancement of antioxidant enzymes in plants	Duo et al., 2018 [57]
*L. sphaericus*	Culture collection	*Meloidogyne incognita*	Chitinase production	Abdel-Salam et al., 2018 [58]
*L. sphaericus*	Pumpkin		P solubilization	Sule et al., 2020 [59]
*Lysinibacillus* sp.			P solubilization, IAA production	Sahu et al., 2018 [60]
*L. fusiformis*	Argentine screwbean	Endophytic bacteria Growth promotion under salt stress conditions	N fixation, IAA production, GA production, ABA production	Sgroyet et al., 2009 [61]
*Lysinibacillus* sp.	Plant	Endophytic bacteria Growth promotion under salt stress conditions	P solubilization, IAA production, extracellular enzymes production	Kumar et al., 2017 [62]
*L. varians*	Waste contaminated soil	Growth promotion under metal stress conditions of Rhizobacteria	Cd and Pb bio-accumulation, NH3production, IAA production, P solubilization, siderophore production	Pal and Sen Gupta, 2019 [63]
*L. fusiformis, L. mangiferihumi,*	Soil		IAA production, K solubilization, siderophore production	Jinal et al., 2019 [64]
*L. fusiformis, L. xylanilyticus*	Forest		P solubilization, IAA production	De Mandal et al., 2018 [65]

## Data Availability

Not applicable.
